# Review of Alzheimer's susceptibility genes in African population

**DOI:** 10.1002/alz70855_107260

**Published:** 2025-12-24

**Authors:** Olatokun Shamsudeen Akano, Halleluyah Darasimi Oludele, Chukwuebuka Stanley Asogwa

**Affiliations:** ^1^ College of Medicine, University of Ibadan, Nigeria., Ibadan, Oyo, Nigeria; ^2^ University of Ibadan, Ibadan, Oyo, Nigeria; ^3^ College of Medicine University of Ibadan, Ibadan, Nigeria; ^4^ University Of Ibadan, Ibadan, Oyo ‐ State, Nigeria; ^5^ College Research and Innovation Hub, Ibadan, Nigeria

## Abstract

**Background:**

In tandem with the rapidly growing aging population in low‐ and middle‐income countries, the burden of dementia, particularly Alzheimer's disease (AD) is escalating in African populations. Despite a two‐fold risk of AD genes in African populations, individuals of African ancestry have been underrepresented in AD genomic research. This limited our ability to identify African causative genetic variants underlying the disease and hamper our efforts in addressing this devastating disease in African populations. Our research emphasizes the need for more studies to identify novel African‐specific genes and fully understand the genetic architecture underlying AD for therapeutic interventions.

**Method:**

We searched PubMed for all papers on genetic studies that have been published in Africa on Alzheimer's disease. 28 relevant papers were selected. The search was carried out using a combination of keywords and phrases such as “Alzheimer's disease,” “genetics,” “susceptibility genes,” “African populations, “European populations.”Inclusion criteria: Peer‐reviewed studies investigating Alzheimer's susceptibility genes in AFRICAN and EUROPEAN populations that have been published. Exclusion criteria: Editorials, commentaries, opinion articles papers without abstracts, and studies with irrelevant methodologies.

**Result:**

The genetic landscape of Alzheimer's disease in African populations reveals distinct patterns, with population‐specific mutations like R35Q in PSEN1 (Mozabite) and V191A (San) contributing to disease risk. The traditional AD‐associated genes (APP, PSEN1, PSEN2) show varied presentations in African populations, particularly in Moroccan cohorts. ABCA7 emerges as a crucial risk factor unique to African populations, while APOE ε4's role remains complex, with conflicting evidence in Yoruba studies. African populations show distinctive genetic signatures through PON1, TREM2, and SLC24A4, emphasizing population‐specific risk factors. Comparing with European populations highlights notable differences, particularly in BIN1 and TREM2 impact, though African genetic studies remain underrepresented at 36% versus 50% for European studies, underscoring the need for expanded African‐focused research.

**Conclusion:**

The results of all the searches conducted on AD have frequently shown a correlation and significant genetic variations between AD genes in African and European populations. The lack of large‐scale African genomic studies highlights an urgent need for more representative research to inform tailored interventions for AD prevention and treatment.

